# Time to diagnosis of symptomatic gastric and oesophageal cancer in the Netherlands: Where is the room for improvement?

**DOI:** 10.1177/2050640620917804

**Published:** 2020-04-06

**Authors:** NF van Erp, CW Helsper, P Slottje, D Brandenbarg, FL Büchner, KM van Asselt, JWM Muris, MF Kortekaas, PHM Peeters, NJ de Wit

**Affiliations:** 1 Julius Centre for Health Sciences and Primary Care, University Medical Centre Utrecht, Utrecht, the Netherlands; 2 Department of General Practice and Elderly Care Medicine, VU University Medical Centre, Amsterdam, the Netherlands; 3 Department of General Practice and Elderly Care Medicine, University of Groningen, Groningen, the Netherlands; 4 Department of Public Health and Primary Care, Leiden University Medical Center, Leiden, the Netherlands; 5 Department of General Practice, Amsterdam UMC, Amsterdam, the Netherlands; 6 Department of Family Medicine, Maastricht University, Maastricht, the Netherlands

**Keywords:** Upper gastrointestinal cancer, oesophageal cancer, gastric cancer, duration, diagnostic pathway, delay, general practice, primary care

## Abstract

**Background:**

An efficient diagnostic pathway and early stage diagnosis for cancer patients is widely pursued. This study aims to chart the duration of the diagnostic pathway for patients with symptomatic oesophageal and gastric cancer, to identify factors associated with long duration and to assess the association of duration with tumour stage at diagnosis.

**Methods:**

This was a retrospective cohort study, using electronic health records of six routine primary care databases covering about 640,000 patients, partly linked to the Netherlands Cancer Registry. Symptomatic patients with oesophageal and gastric cancer (2010–2015) that presented in primary care were included. Duration of four diagnostic intervals was determined: patient interval; first symptoms to primary care consultation, primary care interval; consultation to referral, secondary care interval; referral to diagnosis, and the diagnostic interval; consultation to diagnosis. Characteristics associated with ‘long duration’ (≥P75 duration) were assessed using log-binomial regression. Median durations were stratified for tumour stages.

**Results:**

Among 312 symptomatic patients with upper gastrointestinal cancer, median durations were: patient interval: 29 days (interquartile interval 15–73), primary care interval: 12 days (interquartile interval 1–43), secondary care interval: 13 days (interquartile interval 6–29) and diagnostic interval: 31 days (11–74). Patient interval duration was comparable for patients with and without alarm symptoms. Absence of cancer-specific alarm symptoms was associated with ‘long duration’ of primary care interval and secondary care interval: relative risk 5.0 (95% confidence interval 2.7–9.1) and 2.1 (95% confidence interval 1.3–3.7), respectively. Median diagnostic interval duration for local stage disease was 51 days (interquartile interval 13–135) versus 27 days (interquartile interval 11–71) for advanced stage (*p* = 0.07).

**Conclusion:**

In the diagnostic pathway of upper gastrointestinal cancers, the longest interval is the patient interval. Reducing time to diagnosis may be achieved by improving patients’ awareness of alarm symptoms and by diagnostic strategies which better identify cancer patients despite low suspicion.

## Key summary

### Established knowledge before this study

- Prognosis of oesophageal and gastric cancer is highly dependent on disease stage at diagnosis.
- An efficient diagnostic pathway is key to timely diagnosis.
- To reduce time to diagnosis, more knowledge of interval duration, preventable delay and associations between time to diagnosis and tumour stage is required.

### What are the new findings?

- In the diagnostic pathway of oesophageal and gastric cancer patients in the Netherlands, the patient interval is the longest, with comparable time to presentation in primary care for those with and without alarm symptoms.
- For the majority of patients the median duration of the primary care and secondary care interval is relatively short, especially for those with alarm symptoms, but 10–25% of the patients experience substantially long duration of these intervals.
- Shorter time to diagnosis is seen for those with advanced disease stages, suggesting faster processing for patients with poorer prognosis.
- Collaborative action with clinicians and researchers is needed to improve the diagnostic process, e.g. by developing better test strategies, to better identify patients at risk for cancer, especially among those without alarm symptoms.

## Introduction

Upper gastrointestinal (UGI) cancer, i.e. oesophageal and gastric cancer, has substantial morbidity and mortality rates.^1^ Five-year overall survival rates range from 19–31% in non-metastatic UGI cancer, and for patients with metastatic disease, median overall survival ranges from only 15–25 weeks.^2–5^

One of the explanations of this low level of survival is the fact that UGI cancers are currently diagnosed in a relatively advanced disease stage; 70% of the patients are diagnosed with stage III or IV disease.^6^ This is besides the fact that these types of cancers only become symptomatic in advanced disease stages, and advanced stages may result from delay either before presentation to healthcare services in primary care or during diagnostic work-up in secondary care. According to the literature, shortening the patient interval is probably most vital to reduce delay in the diagnostic pathway of gastroesophageal cancer.^7,8^

In gatekeeper systems like that in the Netherlands, patients have to visit a general practitioner (GP) first and GPs can refer patients to secondary care if needed. Most patients with UGI cancer will therefore initially present with symptoms in primary care. Referral to secondary care is either made urgently (often through telephone contact) or regularly (using a digital referral system). Usually, GPs in the Netherlands have open access to UGI endoscopy, meaning that they can refer patients for this procedure without prior consultation with a gastroenterologist.

Earlier studies reported on the duration of, and factors associated with, delay in different phases of the diagnostic pathway, providing ‘fragmented’ evidence.^8–16^ Delaying factors include symptom recognition and interpretation, patient characteristics and healthcare factors.^9,17^ Although several studies reported on the association between time to diagnosis and tumour stage at diagnosis and/or survival, they considered individual intervals of the diagnostic pathway, hampering solid conclusions.^11,12,18,19^ To improve the diagnostic pathway of UGI cancers, a comprehensive overview of the duration of its intervals and factors contributing to delay is required. The aim of this study is to provide this overview of the duration of the diagnostic pathway for patients with oesophageal and gastric cancer in the Netherlands, to assess characteristics associated with long duration, and to assess the association between duration and tumour stage at diagnosis.

## Methods

### Study design and data source

A retrospective cohort study was performed using anonymised data from six academic general practice networks (Supplementary Material Appendix 1), containing coded and free-text information from primary care electronic health records (EHRs) of over 640,000 patients. Free texts include real-time registrations of patient consultations, i.e. presented complaints, results of physical examination, clinical reasoning of the GP and management plan. This data source was used to determine the duration of the patient interval (IP) and the primary care interval (IPC).

To be able to determine the secondary care interval (ISC), the diagnostic interval (ID) and the association between duration and tumour stage at diagnosis, we linked, where possible, the routine primary care data to the data of the Netherlands Cancer Registry (NCR). The NCR is a population-based registry with detailed diagnostic and therapeutic data of over 95% of Dutch cancer patients since 1989.^20^ Data linkage was possible for three of the six databases (Julius General Practitioner’s Network database (Utrecht) (JGPN), Academic Network of General Practice database (Amsterdam VUmc) (ANH VUmc) and Registration Network Groningen (RNG): together comprising 76% of the cancer patients) as these include pseudonyms based on patient identifiers. Primary care and NCR records were linked based on date of birth, sex and postal code (six digits) among patients with the cancer type in question, using a trusted third-party linkage procedure to comply with privacy regulations of Dutch and International law (General Data Protection Regulation, https://gdpr.eu).

### Case selection

All adult patients (aged ≥18 years) registered with the International Classification of Primary Care (ICPC, version 1)^21^ code for ‘malignant neoplasm of oesophagus’ (D77.01) or ‘malignant neoplasm of stomach’ (D74) in 2010–2015 were extracted from the primary care databases.

Of all identified patients, we checked the free text elements of the EHR to confirm the cancer diagnosis, based on summaries of correspondence from secondary care and other descriptions indicating cancer presence. Only those patients with a confirmed cancer diagnosis were included. Next, we selected only those who presented to the GP with symptoms, and were referred by the GP for diagnostic workup.

### Data collection

Data were collected from the primary care databases and NCR by medically trained researchers (6th year medical students). Primary care EHRs were scrutinised manually from 5 years before the date of entry of the ICPC code for UGI cancer up to 1 year after. EHRs were studied up to 1 year after ICPC coding because the date of the ICPC code marks the beginning of the disease episode and not the actual date of diagnosis as registered in the NCR.

**Figure 1.**
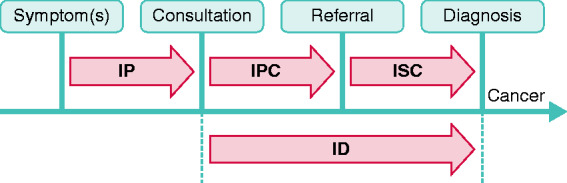
The cancer diagnostic pathway and its intervals, based on the Aarhus statement.^22^ ID: diagnostic interval; IP: patient interval; IPC: primary care interval; ISC: secondary care interval.

Four time intervals of the diagnostic pathway were assessed (Figure 1), based on the definitions provided in the Aarhus statement.^22^ The IP was defined as the time interval between first noticing cancer-related symptom(s) to first consultation for these symptoms in primary care; the IPC was defined as duration from first consultation with cancer-related signs and/or symptoms in primary care to referral to secondary care; the ISC was defined as duration from referral to secondary care by the GP to date of histological diagnosis, and the overarching ID was defined as duration from first consultation to date of diagnosis. Definitions of the different milestones are shown in Table 1.

**Table 1.** Milestones of the diagnostic pathway of symptomatic cancer and their definitions.

	Definition
Date of first symptom(s)	Date of first symptom(s) was defined as registered by the GP in the free-text fields of the electronic health record. If ‘stomach ache since one week’ was registered, date of first symptom was the date 7 days before the date of first consultation. Less strictly described durations, such as ‘several weeks’ and ‘a couple of days’ were interpreted according to predefined rules, Supplementary Material Appendix 2. Duration indications as ‘for a while’ or ‘for some time’ where considered too vague for interpretation and were excluded from IP analysis. In case of different duration indications for multiple cancer related complaints, the longest duration was selected to determine IP duration.
Date of first consultation	Date of first consultation was defined as the first presentation to the GP with signs or symptoms related to the UGI cancer. In case of vague or non-specific signs or symptoms, the first consultation with complaints that eventually led to the cancer diagnosis, and could reasonably be related to the cancer, was taken. We minimised the risk of misattribution of symptoms by discussing doubtful cases in our team of researchers, who are medical doctors with primary care experience.
Date of referral	Date of referral was defined as the moment the responsibility for the patient was transferred from primary to secondary care, as registered in the electronic health record. Referral to radiology or endoscopy department for imaging was considered as referral if abnormal findings subsequently resulted in referral to a specialist, without further interference of the GP. In case of multiple referrals to, or cross-referrals in secondary care, the first referral for further exploration of cancer related symptoms was taken.
Date of diagnosis	To determine ISC and ID duration, the date of diagnosis was retrieved from the NCR for NCR matched patients. The NCR uses the hierarchy for diagnosis date as provided by the European Network of Cancer Registries, primarily registering date of histological diagnosis.

GP: general practitioner; ID: diagnostic interval; IP: patient interval; ISC: secondary care interval; NCR: the Netherlands Cancer Registry; UGI: upper gastrointestinal.

Patient and presentation characteristics were collected from the routine primary care data. All characteristics and methods of collection are shown in Supplementary Material Appendix 3. Symptoms were categorised as UGI cancer-specific alarm symptoms (persistent vomiting, haematemesis or melaena, dysphagia and a palpable mass in the epigastric region),^23^ cancer general alarm symptoms (unintended weight loss, anaemia and ascites) and non-alarming symptoms (all other UGI cancer-related symptoms). Disease characteristics were retrieved from the NCR data for NCR matched patients.

### Analyses

Duration of the four intervals was calculated and stratified for several patient and presentation characteristics and tumour stage at diagnosis. We consistently added one day to all durations, as we considered same-day proceedings as a duration of one day. Differences in median duration were tested with the Mann-Whitney U test for variables with two categories or the Kruskall-Wallis test for variable with ≥3 categories.

To assess associations with ‘long duration’, we defined this as duration equal to or longer than the 75th percentile value (≥P75) of duration for the different intervals (IP, IPC, ISC). Univariable and multivariable log-binomial regression analyses were performed to identify characteristics associated with ‘long duration’. Characteristics that were statistically significantly associated with ‘long duration’ (*p*<0.05) in univariable analysis were included in multivariable analysis, next to age and sex. For IPC, we assessed extra characteristics (consultation frequency, chronic comorbidities and psychiatric comorbidity).

### Software

Data transformation and analyses were performed in SPSS version 22.0 (SPSS Inc., Chicago, Illinois, USA).

### Patient and public involvement

Patients and/or public were not involved in this study.

## Results

### Patient characteristics

**Figure 2.**
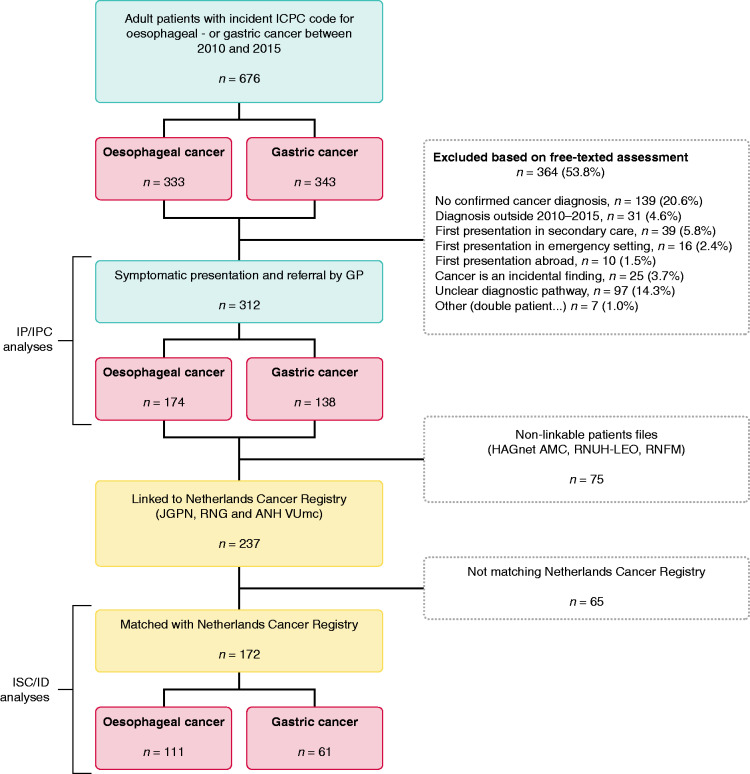
Identified upper gastrointestinal cancer cases and reasons for exclusion. ANH VUmc: Academic Network of General Practice database (Amsterdam VUmc); GP: general practitioner; HAGnet AMC: General Practice Registration Network (Amsterdam AMC); ICPC: International Classification of Primary Care; ID: diagnostic interval; IP: patient interval; IPC: primary care interval; ISC: secondary care interval; JGPN: Julius General Practitioner’s Network database (Utrecht); RNFM: Research Network Family Medicine (Maastricht); RNG: Registration Network Groningen; RNUH-LEO: Registration Network of General Practitioners Associated with Leiden University (Leiden).

Of 676 patients with an ICPC code for oesophageal and gastric cancer, 312 patients (46%) met the eligibility criteria; 174 oesophageal and 138 gastric cancer patients. The most common reasons for exclusion (Figure 2) were a non-confirmed cancer diagnosis (potentially incorrect ICPC code) and an unclear diagnostic pathway (plausible diagnosis but unclear route to diagnosis).

Patient characteristics are described in Table 2. Most of the patients (64%) were male: 70% of the oesophageal cancer patients and 55% of the gastric cancer patients. Mean age at first GP consultation was 66.4 years (standard deviation (SD) 11.9), comparable for oesophageal and gastric cancer. During the first consultation, for around 60% of the patients a cancer-specific alarm symptom was registered: 67% of the oesophageal cancer patients and 54% of the gastric cancer patients.

**Table 2.** Characteristics of patients with upper gastrointestinal (UGI) cancer that presented with symptoms in primary care.

		UGI cancers	Oesophageal cancer	Gastric cancer
Population	*n* (%)	312 (100)	174 (100)	138 (100)
Male patients	*n* (%)	199 (63.8)	123 (70.7)	76 (55.1)
Age at first consultation	Mean ± SD	66.4 ± 11.9	66.6 ± 10.2	66.2 ± 13.8
SES score 2014^a^	Mean ± SD	0.32 ± 1.17	0.39 ± 1.14	0.23 ± 1.22
	Missing, *n* (%)	66 (21.2)	33 (19.0)	33 (23.9)
Consultation frequency in year before first consultation	Median (IQI)	5 (2–10)	5 (2–8)	6 (2–12)
	Missing, *n* (%)	24 (7.7)	9 (5.2)	15 (10.9)
Number of registered chronic somatic comorbidities^b^	Median (IQI)	3 (1–5)	3 (1–6)	3 (1–4)
	Missing, *n* (%)	8 (2.6)	8 (4.6)	0 (0.0)
Registered psychiatric comorbidity^b^	*n* (%)	65 (20.8)	40 (23.0)	25 (18.1)
	Missing, *n* (%)	8 (2.6)	8 (4.6)	0 (0.0)
Dominant symptom(s) at first consultation^c^				
Cancer-specific alarm symptom(s)	*n* (%)	127 (40.7)	86 (49.4)	41 (29.7)
Cancer general alarm symptom(s)	*n* (%)	61 (19.6)	25 (14.4)	36 (26.1)
Other, non-alarming symptoms	*n* (%)	124 (39.7)	63 (36.2)	61 (44.2)
Dominant symptom(s) at referral^c^				
Cancer-specific alarm symptom(s)	*n* (%)	191 (61.2)	117 (67.2)	74 (53.6)
Cancer general alarm symptom(s)	*n* (%)	69 (22.1)	24 (13.8)	45 (32.6)
Other, non-alarming symptoms	*n* (%)	52 (16.7)	33 (19.0)	19 (13.8)
Population linked to NCR^d^	*n* (%)	237 (76.0)	138 (79.3)	99 (71.7)
Match with NCR	n (% of linked)	172 (72.6)	111 (80.4)	61 (61.6)
TNM disease stage at diagnosis				
0, I or II	*n* (% of matched)	42 (24.4)	19 (17.1)	23 (37.7)
III or IV	*n* (% of matched)	122 (70.9)	89 (80.2)	33 (54.1)
Missing	*n* (% of matched)	8 (4.7)	3 (2.7)	5 (8.2)
Morphology				
Adenocarcinoma	*n* (% of matched)	93 (54.1)	57 (51.4)	36 (59.0)
Squamous cell carcinoma	*n* (% of matched)	42 (24.4)	42 (37.8)	–
Other	*n* (% of matched)	37 (21.5)	12 (10.8)	25 (41.0)

ANH VUmc: Academic Network of General Practice database (Amsterdam VUmc); IQI: interquartile interval; JGPN: Julius General Practitioner’s Network database (Utrecht); NCR: the Netherlands Cancer Registry; RNFM: Research Network Family Medicine (Maastricht); RNG: Registration Network Groningen; SD: standard deviation; SES: socio-economic status; TNM: tumour node metastasis.

^a^SES scores of 2014, based on level of education, income and job status. The Dutch mean SES in 2014 was 0.28 (SD 1.09). SES could be derived for patients from four out of the six primary care network databases (JGPN, ANH VUmc, RNG and RNFM).

^b^According to the definitions of O’Halloran et al.^34^

^c^Cancer-specific alarm symptoms for UGI cancers (oesophageal and gastric cancer) were defined as persistent vomiting, UGI bleeding (haematemesis or melaena), dysphagia and a palpable mass in the epigastric region. Cancer-general alarm symptoms were defined as unintended weight loss, anaemia and ascites. Other, non-alarming symptoms were all other presenting symptoms that could be related to the UGI cancer, including abdominal pain, nausea, gastro-oesophageal reflux, malaise etc. In case of presence of both cancer-specific and cancer-general alarm symptoms, cancer-specific alarm symptoms were considered dominant.

^d^Linkage with NCR was possible for three of the six primary care network databases (JGPN, ANH VUmc and RNG).

For the analysis of ISC, ID and the association of duration with tumour stage, a total of 237 patients (76% of eligible) could be linked to the NCR. For 172 patients (73% of those linked) a match was found in the NCR. We found no differences in patient and presentation characteristics between those matching NCR (*n* = 172) and those who did not match (*n* = 65) (Supplementary Material Appendix 4). Of NCR-matched patients, 122 (71%) were diagnosed with advanced disease stage (stage III or IV): 80% among oesophageal cancer patients and 54% among gastric cancer patients.

### Duration of time intervals

**Figure 3.**
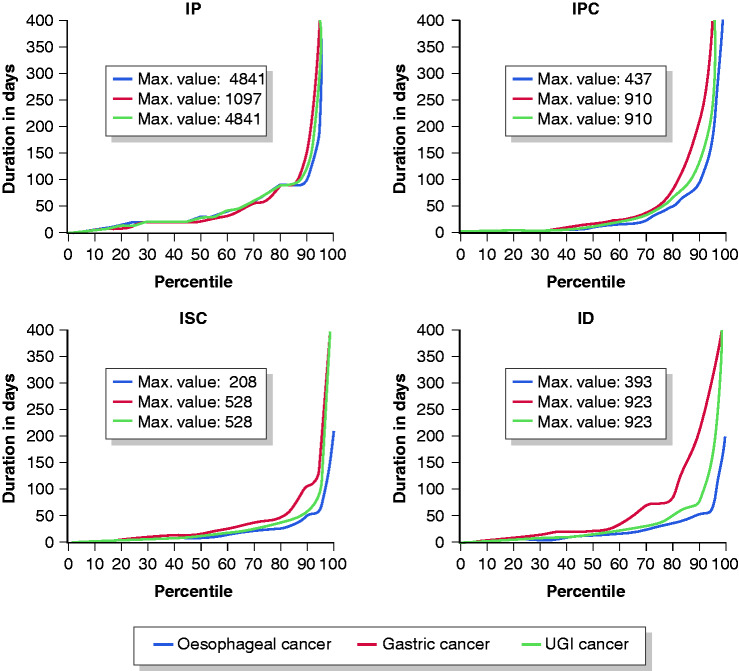
Distribution of the duration of the different intervals of the cancer diagnostic pathway of upper gastrointestinal (UGI) cancer patients. ID: diagnostic interval; IP: patient interval; IPC: primary care interval; ISC: secondary care interval.

Duration of the different intervals is shown in Table 3. All intervals showed a right skewed distribution as shown in Figure 3, with a strong increase in durations for 10–25% of patients with the longest intervals.

**Table 3.** Duration of the intervals of the diagnostic pathway for patients with upper gastrointestinal (UGI) cancer that presented with symptoms in primary care.

	Patient interval			Primary care interval			Secondary care interval^a^			Diagnostic interval^a^		
	*n*	Median (IQI)	*p*-Value^b^	*n*	Median (IQI)	*p*-Value^b^	*n*	Median (IQI)	*p*-Value^b^	*n*	Median (IQI)	*p*-Value^b^
UGI cancers	201	**29 (15–73)**		312	**12 (1–43)**		167	**13 (6–29)**		167	**31 (11–74)**	
Sex												
Men	139	27 (15–75)	0.58	199	8 (1–43)	0.09	109	13 (7–29)	0.73	109	30 (11–67)	0.39
Women	62	29 (22–66)		113	15 (1–45)		58	12 (5–33)		58	32 (12–99)	
Age at first consultation												
<55 years	29	40 (19–95)	0.50	46	23 (2–83)	0.10	22	9 (5–21)	0.32	22	35 (10–102)	0.26
55–64 years	68	28 (17–62)		93	12 (1–46)		49	12 (6–25)		49	22 (9–58)	
65–74 years	60	22 (17–62)		90	10 (1–36)		59	15 (6–29)		59	30 (16–68)	
≥75 years	44	27 (9–77)		83	8 (1–39)		37	18 (7–54)		37	48 (11–131)	
SES 2014^a^												
<National mean	60	31 (22–62)	0.60	97	8 (1–28)	0.19	65	12 (6–31)	0.69	65	35 (12–79)	0.68
≥National mean	87	27 (15–62)		149	13 (1–62)		99	15 (6–29)		99	30 (12–73)	
Dominant symptom(s)^b^												
Specific alarm symp.	94	28 (20–65)	0.14	127	1 (1–12)	<0.01	103	8 (5–24)	0.01	71	13 (5–35)	<0.01
General alarm symp.	33	46 (22–92)		61	11 (3–46)		37	22 (9–67)		32	44 (11–105)	
Other symptom(s)	74	22 (12–62)		124	32 (13–98)		27	15 (7–31)		64	59 (25–138)	
Disease stage at diagnosis												
Stage 0, I or II	23	22 (11–57)	0.19	42	8 (1–50)	0.63	41	20 (7–46)	0.04	41	51 (13–135)	0.07
Stage III or IV	85	31 (22–80)		122	12 (1–33)		119	10 (6–24)		119	27 (11–71)	
Oesophageal cancer	123	**31 (22–76)**		174	**8 (1–38)**		108	**10 (6–24)**		108	**23 (8–60)**	
Sex												
Men	90	31 (15–78)	0.78	123	4 (1–41)	0.24	80	11 (6–26)	0.24	80	22 (8–61)	0.86
Women	33	26 (22–74)		51	15 (1–31)		28	7 (4–24)		28	26 (5–57)	
Age at first consultation												
<55 years	16	36 (15–91)	0.88	20	7 (1–27)	0.92	12	8 (4–16)	0.26	12	15 (5–35)	0.24
55–64 years	47	32 (22–62)		55	12 (1–46)		34	10 (6–23)		34	22 (6–53)	
65–74 years	40	22 (15–69)		57	8 (1–40)		42	9 (5–25)		42	26 (10–69)	
≥75 years	20	31 (22–91)		42	8 (1–38)		20	17 (7–48)		20	41 (7–85)	
SES 2014^a^												
<National mean	36	34 (22–73)	0.70	53	3 (1–21)	0.06	38	8 (5–19)	0.29	38	18 (6–52)	0.14
≥National mean	56	31 (15–72)		88	11 (1–52)		67	12 (6–28)		67	27 (11–68)	
Dominant symptom(s)^b^												
Specific alarm symp.	66	30 (22–78)	0.02	86	1 (1–13)	<0.01	74	8 (5–20)	0.04	74	20 (6–55)	<0.01
General alarm symp.	13	71 (37–106)		25	11 (4–68)		16	22 (6–58)		16	63 (7–105)	
Other symptom(s)	44	22 (9–55)		63	23 (10–67)		18	19 (9–32)		18	31 (17–52)	
Disease stage at diagnosis												
Stage 0, I or II	12	25 (14–59)	0.44	19	3 (1–21)	0.56	18	23 (6–40)	0.12	18	35 (7–64)	0.60
Stage III or IV	67	32 (22–76)		89	4 (1–26)		87	9 (5–22)		87	22 (8–60)	
Gastric cancer	78	**25 (15–62)**		138	**14 (1–51)**		59	**16 (8–42)**		59	**44 (20–145)**	
Sex												
Men	49	22 (13–62)	0.50	76	14 (1–43)	0.47	29	15 (8–39)	0.92	29	44 (30–135)	0.87
Women	29	29 (18–70)		62	15 (2–69)		30	17 (7–44)		30	46 (15–209)	
Age at first consultation												
<55 years	13	49 (22–110)	0.27	26	40 (16–130)	0.01	10	16 (7–41)	0.95	10	114 (35–411)	0.25
55–64 years	21	22 (15–93)		38	13 (1–47)		15	13 (9–47)		15	31 (10–138)	
65–74 years	20	24 (22–56)		33	13 (1–31)		17	16 (8–37)		17	37 (24–71)	
≥75 years	24	22 (4–53)		41	8 (1–40)		17	20 (6–89)		17	79 14–149)	
SES 2014^c^												
<National mean	24	29 (15–62)	0.66	44	17 (1–43)	0.95	27	17 (8–43)	0.69	27	63 (35–171)	0.05
≥National mean	31	22 (11–61)		61	14 (1–79)		32	15 (7–37)		32	35 (13–144)	
Dominant symptom(s)^d^												
Specific alarm symp.	28	23 (3–59)	0.51	41	1 (1–12)	<0.01	29	15 (6–37)	0.13	14	33 (8–40)	<0.01
General alarm symp.	20	32 (21–85)		36	11 (1–36)		21	21 (10–89)		17	43 (16–130)	
Other symptom(s)	30	22 (14–93)		61	40 (16–170)		9	14 (6–27)		28	109 (27–334)	
Disease stage at diagnosis												
Stage 0, I or II	11	22 (8–32)	0.44	23	18 (2–130)	0.91	23	20 (9–47)	0.47	23	63 (20–171)	0.52
Stage III or IV	18	24 (20–94)		33	21 (10–74)		32	16 (8–37)		32	41 (21–136)	

ANH VUmc: Academic Network of General Practice database (Amsterdam VUmc); IQI: interquartile interval; JGPN: Julius General Practitioner’s Network database (Utrecht); RNFM: Research Network Family Medicine (Maastricht); RNG: Registration Network Groningen; SD: standard deviation; SES: socio-economic status.

Specific alarm symp.: cancer-specific alarm symptom(s), general alarm symp.=cancer general alarm symptom(s).

^a^Four patients with negative secondary care interval durations were excluded from secondary care- and diagnostic interval analysis.

^b^Differences in median duration were tested with a Mann-Whitney U test (two categories) or a Kruskall-Wallis test (≥3 categories).

^c^SES scores of 2014, based on level of education, income and job status. The Dutch mean SES in 2014 was 0.28 (SD 1.09). SES could be derived for patients from four out of the six primary care network databases (JGPN, ANH VUmc, RNG and RNFM).

^d^Cancer-specific alarm symptoms for UGI cancers (oesophageal and gastric cancer) were defined as persistent vomiting, UGI bleeding (haematemesis or melaena), dysphagia and a palpable mass in the epigastric region. Cancer general alarm symptoms were defined as unintended weight loss, anaemia and ascites. Other, non-alarming symptoms were all other presenting symptoms that could be related to the UGI cancer, including abdominal pain, nausea, gastro-oesophageal reflux, malaise etc. In cases of presence of both cancer-specific and cancer general alarm symptoms, cancer-specific alarm symptoms were considered dominant. For the patient, primary care and diagnostic intervals, symptoms at first consultation were used, for the secondary care interval, symptoms as present at referral were used.

An IP) was reported for 201 patients (64%). It could not be determined for 29% and 43% of oesophageal and gastric cancer patients, respectively. The median duration of IP was 29 days (interquartile interval (IQI) 15–73), 31 days (IQI 22–76) for oesophageal cancer and 25 days (IQI 15–62) for gastric cancer. Although statistically non-significant, longer IP durations were seen for younger patients. Patients without alarm symptoms had the shortest median IP duration (22 days (IQI 12–62)), those with general cancer alarm symptoms the longest (46 days (IQI 22–92)).

The median duration of the IPC was 12 days (IQI 1–43), it was 8 days (IQI 1–38) for oesophageal cancer and 14 days (IQI 1–51) for gastric cancer patients. Although statistically non-significant, women had a longer duration of 15 days (IQI 1–45) as compared to 8 days (IQI 1–43) for men. The shortest durations were seen for patients with UGI-specific cancer-alarm symptoms: 1 day (IQI 1–12), as compared to 11 days (IQI 3–46) and 32 days (IQI 13–98) for patients with general cancer alarm symptoms and patients without alarm symptoms, respectively (*p*<0.01). For gastric cancer, patients under 55 years showed statistically significant longer median duration to referral of 40 days (IQI 16–130) as compared to 8 days (IQI 1–40) for patients aged 75 years and older, *p* = 0.01.

The median duration of the ISC was 13 days (IQI 6–29), with shortest durations for those with cancer-specific alarm symptoms (8 days, IQI 5–24) (Table 2). Median duration of the ID was 31 days (IQI 11–74): 23 days for oesophageal cancer (IQI 8–60) and 44 days (IQI 20–145) for gastric cancer. Patients with UGI cancer-specific alarm symptoms showed the shortest ID durations (Table 2). Four patients, who showed negative durations of the ISC, suggesting registration errors, were excluded from ISC and ID analyses.

Results of the log-binomial regression analyses for association with ‘long duration’ (≥P75) of the respective intervals are shown in Table 4. Please note; the absolute number of days that the 75th percentile (cut-off for ‘long duration’) represents, differs for each interval. In short: for IP, no characteristics were found to be statistically significantly associated with ‘long duration’. For IPC, patients without cancer-specific alarm symptoms showed a higher risk for ‘long duration’ in multivariable analysis (RR 5.0, 95% CI 2.7–9.1). For ISC, patients with cancer general alarm symptoms showed a higher risk for ‘long duration’ in multivariable analysis (RR 2.1, 95% CI 1.3–3.7).

**Table 4.** Log-binomial regression analyses for association with ‘long duration’ (≥P75) for the different intervals of the diagnostic pathway, for patients with upper gastrointestinal (UGI) cancer that presented with symptoms in primary care.

	Patient interval				Primary care interval				Secondary care interval^a^			
	≥73 days				≥43 days				≥29 days			
UGI cancers	Univariable RR (95% CI)	*p*-Value	Multivariab. RR (95% CI)	*p*-Value	Univariable RR (95% CI)	*p*-Value	Multivariab. RR (95% CI)	*p*-Value	Univariable RR (95% CI)	*p*-Value	Multivariab. RR (95% CI)	*p*-Value
Sex												
Men	Ref.				Ref.		Ref.		Ref.		Ref.	
Women	1.0 (0.6–1.6)	0.88	–	–	1.0 (0.7–1.5)	0.92	0.9 (0.6–1.3)	0.47	1.2 (0.7–2.0)	0.52	1.1 (0.7–1.8)	0.79
Age at first consultation												
<55 years	Ref.				Ref.		Ref.		Ref.		Ref.	
55–64 years	0.7 (0.4–1.3)	0.26	–	–	0.8 (0.5–1.4)	0.48	0.9 (0.6–1.5)	0.80	1.2 (0.4–3.5)	0.69	1.2 (0.4–3.3)	0.70
65–74 years	0.6 (0.3–1.2)	0.13	–	–	0.7 (0.4–1.2)	0.19	0.7 (0.4–1.2)	0.15	1.4 (0.5–3.8)	0.51	1.3 (0.5–3.5)	0.59
≥75 years	0.8 (0.4–1.6)	0.51	–	–	0.7 (0.4–1.2)	0.23	0.8 (0.5–1.4)	0.43	2.1 (0.8–5.5)	0.14	2.0 (0.8–5.1)	0.16
SES 2014^b^												
<National mean	Ref.				Ref.				Ref.			
≥National mean	0.9 (0.5–1.7)	0.83	–	–	1.5 (0.9–2.4)	0.09	–	–	1.0 (0.6–1.8)	0.88	–	–
Consultation frequency												
<3	n/a	n/a	n/a	n/a	Ref.				n/a	n/a	n/a	n/a
3–6	n/a	n/a	n/a	n/a	0.9 (0.5–1.5)	0.70	–	–	n/a	n/a	n/a	n/a
≥7	n/a	n/a	n/a	n/a	1.1 (0.7–1.8)	0.64	–	–	n/a	n/a	n/a	n/a
Chronic comorbidities^c^												
<2	n/a	n/a	n/a	n/a	Ref.				n/a	n/a	n/a	n/a
2–5	n/a	n/a	n/a	n/a	1.0 (0.7–1.7)	0.86	–	–	n/a	n/a	n/a	n/a
≥6	n/a	n/a	n/a	n/a	1.2 (0.7–2.1)	0.44	–	–	n/a	n/a	n/a	n/a
Psychiatric Comorbidity^c^												
None	n/a	n/a	n/a	n/a	Ref				n/a	n/a	n/a	n/a
≥1	n/a	n/a	n/a	n/a	1.0 (0.6–1.6)	0.88	–	–	n/a	n/a	n/a	n/a
Dominant symptom(s)^d^												
Specific alarm symp.	Ref.				Ref.		Ref.		Ref.		Ref.	
General alarm symp.	1.4 (0.7–2.5)	0.31	–	–	3.0 (1.5–6.1)	<0.01	3.1 (1.5–6.3)	<0.01	2.2 (1.3–3.8)	<0.01	2.1 (1.3–3.7)	<0.01
Other symptom(s)	0.9 (0.5–1.5)	0.67	–	–	4.8 (2.7–8.8)	0.00	5.0 (2.7–9.1)	0.00	1.5 (0.8–3.1)	0.24	1.6 (0.8–3.2)	0.22

ANH VUmc: Academic Network of General Practice database (Amsterdam VUmc); CI: confidence interval.; JGPN: Julius General Practitioner’s Network database (Utrecht); RNFM: Research Network Family Medicine (Maastricht); RNG: Registration Network Groningen; RR; relative risk; SD: standard deviation; SES: socio-economic status.

General alarm symp.=cancer general alarm symptom(s), multivariab.=multivariable, specific alarm symp.=cancer-specific alarm symptom(s).

^a^Four patients with negative secondary care interval durations were excluded from secondary care interval analysis.

^b^SES scores of 2014, based on level of education, income and job status. The Dutch mean SES in 2014 was 0.28 (SD 1.09). SES could be derived for patients from four out of the six primary care network databases (JGPN, ANH VUmc, RNG and RNFM).

^c^According to the definitions of O’Halloran et al.^34^

^d^Cancer-specific alarm symptoms for UGI cancers (oesophageal and gastric cancer) were defined as persistent vomiting, UGI bleeding (haematemesis or melaena), dysphagia and a palpable mass in the epigastric region. Cancer general alarm symptoms were defined as unintended weight loss, anaemia and ascites. Other, non-alarming symptoms were all other presenting symptoms that could be related to the UGI cancer, including abdominal pain, nausea, gastro-oesophageal reflux, malaise etc. In case of presence of both cancer-specific and cancer general alarm symptoms, cancer-specific alarm symptoms were considered dominant. For the patient- and primary care interval, symptoms at first consultation were used, for the secondary care interval, symptoms as present at referral were used.

### Association of duration with tumour stage at diagnosis

For NCR-matched patients (*n* = 172), duration of the respective intervals according to disease stage are shown in Table 3. Median IP and IPC durations were shorter (though not statistically significant) for patients with localised disease (stage 0, I or II) as compared to patients with advanced disease (stage III and IV). Median ISC duration was longer (20 days, versus 10 days, *p*-value: 0.04) for patients with local disease as compared to patients with advanced disease stage. Median ID duration was almost twice as long for those with local disease as compared to patients with advanced disease stage (51 days, versus 27 days, *p*-value: 0.07). At first GP consultation, 54 of 122 (44.3%) patients with advanced disease stage had cancer-specific alarm symptoms, as compared to 15 of 42 patients (35.7%) with localised disease (Supplementary Material Appendix 5).

## Discussion

### Summary of the main findings

In the diagnostic pathway of patients with UGI cancer, the IP is the longest. Median IP duration was 29 days and comparable for patients with and without alarm symptoms. Intervals in both primary and secondary care were relatively short, with a median duration of 12 and 13 days respectively. The median duration of the overall ID was 31 days; 23 days for oesophageal cancer and 44 days for gastric cancer. In all intervals, 10–25% of the patients showed a relatively long duration. Absence of cancer-specific alarm symptoms was associated with ‘long duration’ (≥P75) for both IPC and ISC. We found shorter durations of ISC and ID for patients with advanced disease stages.

### Strengths and limitations

Strengths and limitations of the use of routine primary care data have previously been discussed elsewhere.^24^ The main strength of the current dataset is the availability of free-text annotations of all GP consultations, as this provides detailed insight in the diagnostic process, including GP considerations and contextual factors. We chose not to censor the length of any of the intervals at a maximum time period, as done in previous studies, as the free-text registrations confirmed that some interval durations are very long for plausible reasons. Furthermore, linkage of primary care data to a national cancer registry (NCR), allowed us to analyse all intervals of the diagnostic pathway in one study.

Limitations include the number of excluded patients. This includes patients for whom the ICPC code for UGI cancer was not supported by the free-text registrations (20% of UGI cancer ICPC codes). Reasons for not being able to verify these diagnoses varied from lacking information to clearly incorrect use of the ICPC code (e.g. cancer diagnostic code used for a positive family history of cancer or for other UGI complaints). It has been shown earlier that, when cancer registry-based validation is performed, up to half of the ICPC codes for cancer in primary care records turn out to be incorrectly assigned (‘false positive').^25^ As we were not able to link all patients to the NCR for diagnostic confirmation, we choose to strictly include only those patients for whom the free text of the primary care record confirmed the UGI cancer diagnosis. Furthermore, we excluded patients with unclear diagnostic pathways (14%) and those presenting in emergency settings (2%). This may have affected our findings as, for example, unclear pathways may be more likely for very short or very long diagnostic intervals. Also, patients diagnosed in emergency settings may include patients that could have been referred from primary care and may have more had advanced tumour stages.^26^

We were able to link 76% of eligible patients to the NCR, enabling ISC and ID duration assessment. For the remaining 24% of patients linkage was not possible, because some of the primary care databases used did not contain the right pseudonyms for data-linkage (pseudonym based on postal code, birthdate and sex). As we used the primary care record to verify the UGI cancer diagnosis, we were quite certain of the presence of cancer. However, of the patients for whom linkage could be performed, not all patients (73%) matched with the NCR. We hypothesise that the main reasons for not matching the NCR were changes of postal codes (patients who moved between registry at GP and at registration in NCR) and typographic errors. Even though matching and non-matching patients did not differ substantially with respect to patient- and presentation characteristics, ‘non-matching’ may have been not random, e.g. in cases of ‘patients with changing postal-codes’.

Furthermore, identifying the first presentation with cancer-related symptoms in open-text fields of primary care data is challenging, especially in cases of vague or less specific symptoms. Even though our approach has limitations, we believe it is more accurate than the sole use of diagnostic codes or retrospective questionnaires to identify a first presentation. Free text availability enables the retrieval of a broad range of potential first symptoms, registered at the time of occurrence, which can be extracted from a larger body of daily care registrations. We minimised the risk of misattribution of symptoms by discussing doubtful cases in our team of researchers with primary care experience.

Accurate measurement of the patient interval is known to be challenging and the methods we used come with some limitations.^22,27,28^ The registration of symptom duration in the EHR is a reflection of the GP’s interpretation of the duration that the patient remembered and mentioned. Inaccurate or lacking registration may occur and missing duration information is potentially selective, as doctors may be more prone to register either remarkably short or long durations. We found 29% and 43% missing patient intervals among oesophageal and gastric cancer patients, respectively. Less specific registrations of IP durations also occurred, for which we used a standardised approach to approximate duration (definitions in Supplementary Material Appendix 2). Therefore, whereas IPC, ISC and ID duration should be trusted to the day, IP medians should be seen as an approximation of duration.

### Comparison with existing literature and implications

We found longer median IP durations than earlier reports in the UK, that described median durations of 21.5 days (IQI 7–46) for oesophageal cancer and 9 days (IQI 0–38) for gastric cancer.^8,13^ Even though previous studies suggest that patients consult the GP earlier when their symptoms are more serious (like pain or bleeding),^9^ our findings indicate that patients may not be fully aware of alarm symptoms, since durations of the patient interval for patients with and without registered alarm symptoms were comparable. We believe that raising patients’ awareness of UGI cancer alarm symptoms may be the most efficient way to improve prompt presentation and shorten time to diagnosis. Getting more insight in reasons for postponing consultation would be required for a targeted approach.

Previously reported median durations of IPC range from 1 day (IQI 0–32) for oesophageal cancer^16^ to 12 days for gastric cancer (IQI 0–65);^13^ some were slightly shorter than the IPC durations we found. The main factor earlier reported to be associated with ‘delay’ in primary care is an ‘initial misdiagnosis’.^9^ Even though this sounds as an avoidable and even blameworthy reason for delay, it may be seen as a reflection of risk assessment and the gatekeeping role of the GP. Our finding that absence of alarm symptoms was associated with ‘long duration’ in primary care is in line with this. Improving timely detection of cancer among patients without alarm symptoms is challenging, given the high incidence of common UGI symptoms and low risk of cancer.^29^ Simply lowering the threshold for referral is not the solution for reducing time to referral: apart from the increasing risk of non-indicated endoscopies with normal results, there is already a growing demand for diagnostic services in secondary care. We believe that development of novel diagnostic strategies for patients with less-specific symptoms in primary care is needed, either based on improved selection of patients at risk (for example by decision support tools derived through artificial intelligence in big databases), on the application of diagnostic tests (like the cytosponge for Barrett’s oesophagus, presently evaluated in the UK) or on the use of new biomarkers for gastric and oesophageal cancer.^30^ Since 10–25% of the patients show a strong increase in time to referral, there also is a need for in-depth exploration of the reasons for very long primary care intervals.

Compared to previous UK studies, we found shorter or comparable median durations of ID. Din et al. reported median ID durations of 83 days (IQI 35–207) and 84 days (IQI 35–199) for oesophageal and gastric cancer respectively,^14^ while Swann et al. reported comparable durations of 28 days (IQI 12–66) and 42 days (IQI 17–89).^16^ Even though these differences may be partly explained by different research methods used, they probably reflect true and notable differences in ID durations between different healthcare systems, societies and time periods. This deserves further international comparison, since it could provide clues for reducing the time to diagnosis.

Whether reduction of the duration of the intervals in the diagnostic pathway would improve clinical outcomes is uncertain. Some earlier studies showed that increased durations of ID were associated with advanced disease stage or worse clinical outcomes.^19,31^ In contrast, we found longer durations of both ISC and ID, for patients diagnosed with local disease stage (stage 0, I or II). As slightly more patients with advanced disease stage had specific alarm symptoms, we believe that for the majority of patients this reflects an adequately functioning healthcare system, with quick response for those who are most in need. This concept; long duration for early stage disease, is known as the ‘waiting time paradox’.^32^ Truly understanding the association between time to diagnosis and stage at diagnosis is complex. It has been shown before that the association between waiting times and disease stage or clinical outcomes is not simply linear and that observational studies are not the ideal design for assessment of this association.^33^ More refined methodology is required to enable future studies to unravel the complex association between duration and tumour stage for these cancer types.

## Conclusion

In the diagnostic pathway of UGI cancers, the longest interval is the IP, equally long for patients with and without cancer alarm symptoms. A relatively short ID, especially for those with alarm symptoms and those with advanced disease, suggests faster processing for the sickest patients. Durations of the IPC and ISC are generally acceptable, but nonetheless, remarkably long for 10–25% of the cancer patients. Apart from improving patients’ awareness of alarm symptoms, further reduction of delay in diagnosing UGI cancer may be feasible by introducing novel diagnostic strategies for cancer patients with gastrointestinal symptoms who are currently considered at low risk because of ‘low suspect’ clinical presentation.

## Supplemental Material

UEG917804 Supplemental Material - Supplemental material for Time to diagnosis of symptomatic gastric and oesophageal cancer in the Netherlands: Where is the room for improvement? Click here for additional data file. Supplemental material, UEG917804 Supplemental Material for Time to diagnosis of symptomatic gastric and oesophageal cancer in the Netherlands: Where is the room for improvement? by NF van Erp, CW Helsper, P Slottje, D Brandenbarg, FL Büchner, KM van Asselt, JWM Muris, MF Kortekaas, PHM Peeters and NJ de Wit in United European Gastroenterology Journal
