# Supplementary material for: Time to diagnosis of symptomatic gastric and oesophageal cancer in the Netherlands: Where is the room for improvement?
Source: United European Gastroenterol J. 2020 Apr 6;8(5):607–20. doi: 10.1177/2050640620917804 (PMC7268938; doi:10.1177/2050640620917804)
Supplement: UEG917804 Supplemental Material - Supplemental material for Time to diagnosis of symptomatic gastric and oesophageal cancer in the Netherlands: Where is the room for improvement? [file UEG917804_Supplemental_Material.pdf]

Appendix 1. Description of the six Dutch primary care network databases.

JGPN (Utrecht)

The Julius General Practitioner's Network (JGPN) database contains free-text and coded information from primary care electronic health records (EHRs) of over 300,000 patients, subscribed to 52 GP practices in a central region of the Netherlands.[35]

ANH VUmc (Amsterdam)

The Database of the Academic Network of General Practice, Department of General Practice & Elderly Care Medicine VU University Medical Centre (ANH VUmc) is a longitudinal database containing pseudonimyzed coded and free text data extracted from the EHR of around 197,000 patients enlisted in about 50 practices in the Amsterdam region in the studied time period. Free text data are anonymized before they are used by researchers.

RNG (Groningen)

Registration Network Groningen (RNG) comprises of three GP group practices in the northern part of the Netherlands and has a dynamic patient population of approximately 30,000 patients. No free-text is available in the database, medical records were studied on location in the participating GP-practices. In this database, patients with ICPC-1 code D77 ('malignant neoplasm digestive organs other/NOS') instead of D77.01 were initially selected as no subcodes were specified in this database.

HAGnet AMC (Amsterdam)

The general practice registration network database of the Department of General Practice, Academic Medical Center Amsterdam, contains routinely collected free text and coded data of 46,000 patients of 49 GPs working at six primary care centres.

RNUH-LEO (Leiden)

Registration Network of General Practitioners Associated with Leiden University (RNUH-LEO) is a longitudinal database containing electronic health records of over 40,000 patients subscribed to 19 GP practices organised in 4 healthcare centres in the western region of the Netherlands. Since 2016, RNUH-LEO is transformed into Extramural Leiden Academic Network (ELAN) with data from GP and patients in the Leiden and The Hague area. Website: https://www.lumc.nl/org/pheg/research/collaboration/elan-extramuraal-leids-academisch-netwerk/?setlanguage=English&setcountry=en

RNFM (Maastricht)

The Research Network Family Medicine (RNFM) is a continuous database in which about 70 GPs working in 22 different practices in the South of the Netherlands are participating. For this study, data of 8 practices with a total

population of almost 28,000 patients were included. At the time of data-extraction, no free-text was available in the database, therefore medical records were studied on location in the participating GP-practices. Website: https://www.rnfm.nl

Appendix 2. Duration of symptoms as recorded in primary care records: interpretation and rules for date registration.

	GP registry of duration	Interpretation	Rule for date registration
**Range**	Since 2-3 months/years	Mean; 2.5 months/years	Halfway through month: 15th (in February: 14th) Halfway through year: first of july
**Acute**	Since last night	0 days	Date of consultation
**Days**	Couple/few/several days	3 days	3 days before date of consultation
	Since last weekend	Saturday as reference day	Saturday before date of consultation
**Weeks**	Since last week	7 days	7 days before date of consultation
	For over a week	9 days	9 days before date of consultation
	Since the end of last week	Friday as reference day	Friday before date of consultation
	For a week and a half	11 days	11 days before date of consultation
	Couple/few/several weeks	3 weeks	21 days before date of consultation
**Months**	Since one month	1 calender month	Same date, one month before
	For over a month	1 month and one week	Same date, one month before, minus 7 days
	For over two months	2.5 month	Halfway through month: 15th (in February: 14th)
	Since december	Halfway through the month	Halfway through month: 15th (in February: 14th)
	Since the end of december	Since the last day of the month	30th or 31th (in February: 28th or 29th)
	Couple/few/several months	3 calendar months	Same date, three months before
**Years**	Since years	3 calendar years	Same date, three years before
	Couple/few/several years	3 calendar years	Same date, three years before
	Since over a year	1 year and three months	Same date one year and three calendar months before
	Since the beginning of the year	Since january first	January first of that year
	Since the year … (eg 2008)	Halfway through year	Halfway through year: first of july
**Vague**	Since a while	Too vague, no interpretation	None
	For some time		
	For a long time		
	Etc.		

Appendix 3. Characteristics and methods of collection.

Characteristic	Method of collection
**Sex**	Sex was extracted from the routine primary care databases, in which this is registered for all patients.
**Age at first consultation**	Age was calculated based on birthyear as registered in the routine primary care databases. For all patients, July first was set as their birthdate as only year of birth was available for analysis. Age at first consultation was then calculated. Age was categorized as a non-linear association with duration of the primary care interval was expected
**Socio-economic status score (SES) 2014**	SES was retrieved from publicly available data from the Netherlands Institute for Social Research, in which status scores are available according to 4-digit postal code and based on level of education, income and job status. The scores of 2014 were used and attached to the dataset by the datamanagers of the respective routine primary care databases. The Dutch mean SES in 2014 was 0.28 (SD 1.09). Patients were divided in two categories based on the Dutch mean: SES < 0.28 and SES ≥ 0.28.
**Consultation frequency in year before first consultation**	We used the consultation frequency in the year preceding first consultation as a measure of how frequent a patients generally visits the general practitioner, in other words: to identify 'frequent visitors'. Number of GP consultations in the year before the first cancer related consultation was determined by counting all registered physical or phone contacts with the practice, except for repeated prescriptions and registered correspondence with secondary care.
**Number of registered chronic somatic comorbidities and registered psychiatric comorbidity**	Episode lists in the EHRs were used to determine existence of (chronic) comorbidities. To decide on relevance and chronicity of registered episodes, the list of chronic comorbidities in primary care as provided by O'Halloran et al. was used as guidance. In this list included ICPC-codes starting with a "P" were regarded as relevant psychiatric comorbidities.
**Dominant symptom(s) at first consultation**	Information on symptoms at first consultation was retrieved from the free text consultation registries in the routine primary care databases. Cancer specific alarm symptoms for UGI cancers (oesophageal and gastric cancer) were defined as persistent vomiting, UGI bleeding (hematemesis or melena), dysphagia and a palpable mass in the epigastric region. Cancer general alarm symptoms were defined as unintended weight loss, anaemia and ascites. Other, non-alarming symptoms were all other presenting symptoms that could be related to the UGI-cancer, including abdominal pain, nausea, gastro-oesophageal reflux, malaise etc. In case of presence of both cancer specific and cancer general alarm symptoms, cancer specific alarm symptoms were considered dominant.
**Dominant symptom(s) at referral**	Information on symptoms at referral was retrieved from the free text consultation registries in the routine primary care databases. Dominant symptoms at referral included the dominant symptom(s) that occurred somewhere after first consultation and before referral. In case of presence of both cancer specific and cancer general alarm symptoms, cancer specific alarm symptoms were considered dominant.
**Disease stage at diagnosis and tumour morphology**	Disease stage at diagnosis and tumour morphology were extracted from the NCR database. In the NCR, tumour stage is registered according to the stage grouping of the Tumour Node Metastasis (TNM) system.

GP = General Practitioner, EHR = Electronic Health Record, UGI cancer = Upper Gastrointestinal cancer, NCR = the Netherlands Cancer Registry.

Appendix 4. For symptomatic upper gastrointestinal cancer patients that could be linked to the Netherlands Cancer Registry: characteristics of patients that were a match with NCR compared with patients that were not a match with NCR.

		**NCR match**	**No match**
**Population**	n (%)	172 (100)	65 (100)
**Male patients**	n (%)	111 (64.5)	39 (60.0)
**Age at first consultation**	Mean ± SD	65.9 ± 11.8	67.5 ± 13.7
**Socio-economic status score (SES) 2014**[a]	Mean ± SD	0.36 ± 1.16	0.43 ± 1.14
	Missing, n (%)	3 (1.7)	2 (3.1)
**Consultation frequency in year before first consultation**	Median (IQI)	5 (2-11)	5 (2-9)
	Missing, n (%)	14 (8.1)	9 (13.8)
**Number of registered chronic somatic comorbidities** [b]	Median (IQI)	3 (1-6)	2 (1-4)
	Missing, n (%)	6 (3.5)	2 (3.1)
**Registered psychiatric comorbidity**[b]	n (%)	40 (23.3)	8 (12.3)
	Missing, n (%)	6 (3.5)	2 (3.1)
**Dominant symptom(s) at first consultation**[c]			
Cancer specific alarm symptom(s)	n (%)	72 (41.9)	27 (41.5)
Cancer general alarm symptom(s)	n (%)	32 (18.6)	13 (20.0)
Other, non-alarming symptoms	n (%)	68 (39.5)	25 (38.5)
**Dominant symptom(s) at referral**[c]			
Cancer specific alarm symptom(s)	n (%)	106 (61.6)	38 (58.5)
Cancer general alarm symptom(s)	n (%)	38 (22.1)	15 (23.1)
Other, non-alarming symptoms	n (%)	28 (16.3)	12 (18.5)

IQI = interquartile interval, NCR = Netherlands Cancer Registry, SD = standard deviation
[a] Socio-economic status scores of 2014, based on level of education, income and job status. The Dutch mean SES in 2014 was 0.28 (SD 1.09). SES could be derived for patients from 4 out of the 6 primary care network databases (JGPN, ANH VUmc, RNG and RNFM).
[b] According to the definitions of O'Halloran et al.[31]
[c] Cancer specific alarm symptoms for UGI cancers (oesophageal- and gastric cancer) were defined as persistent vomiting, UGI bleeding (hematemesis or melena), dysphagia and a palpable mass in the epigastric region. Cancer general alarm symptoms were defined as unintended weight loss, anaemia and ascites. Other, non-alarming symptoms were all other presenting symptoms that could be related to the UGI cancer, including abdominal pain, nausea, gastro-oesophageal reflux, malaise etc. In case of presence of both cancer specific and cancer general alarm symptoms, cancer specific alarm symptoms were considered dominant.

Appendix 5. For symptomatic upper gastrointestinal cancer patients with available disease stage: symptom distribution according to tumour stage at diagnosis and different time intervals.

		**Patient interval**	**Primary care interval**	**Secondary care interval***	**Diagnostic interval***
Disease stage	Dominant symptom(s)[a]	n (%)	n (%)	n (%)	n (%)
**Stage 0, I or II**	*All*	23 (100)	42 (100)	41 (100)	41 (100)
	Specific alarm symp.	11 (47.8)	15 (35.7)	19 (46.3)	14 (34.1)
	General alarm symp.	5 (21.7)	11 (26.2)	14 (34.1)	11 (26.8)
	Other symptom(s)	7 (30.4)	16 (38.1)	8 (19.5)	16 (39.0)
**Stage III or IV**	*All*	85 (100)	122 (100)	119 (100)	119 (100)
	Specific alarm symp.	43 (50.6)	54 (44.3)	81 (68.1)	54 (45.4)
	General alarm symp.	10 (11.8)	17 (13.9)	19 (16.0)	17 (14.3)
	Other symptom(s)	32 (37.6)	51 (41.8)	19 (16.0)	48 (40.3)

General alarm symp. = cancer general alarmsymptom(s), Specific alarm symp. = cancer specific alarm symptom(s).
[a] Cancer specific alarm symptoms for UGI cancers (oesophageal- and gastric cancer) were defined as persistent vomiting, UGI bleeding (hematemesis or melena), dysphagia and a palpable mass in the epigastric region. Cancer general alarm symptoms were defined as unintended weight loss, anaemia and ascites. Other, non-alarming symptoms were all other presenting symptoms that could be related to the UGI cancer, including abdominal pain, nausea, gastro-oesophageal reflux, malaise etc. In case of presence of both cancer specific and cancer general alarm symptoms, cancer specific alarm symptoms were considered dominant. For the patient-, primary care and diagnostic interval, symptoms at first consultation were used, for the secondary care interval, symptoms as present at referral were used. *Four patients with negative secondary care interval durations were excluded from secondary care- and diagnostic interval analysis

STROBE Statement—Checklist of items that should be included in reports of ***cohort studies***

	Item No	Recommendation	Page No
**Title and abstract**	1	(*a*) Indicate the study's design with a commonly used term in the title or the abstract<br>(*b*) Provide in the abstract an informative and balanced summary of what was done and what was found	1-2<br>2
**Introduction**			
Background/rationale	2	Explain the scientific background and rationale for the investigation being reported	4
Objectives	3	State specific objectives, including any prespecified hypotheses	4
**Methods**			
Study design	4	Present key elements of study design early in the paper	5
Setting	5	Describe the setting, locations, and relevant dates, including periods of recruitment, exposure, follow-up, and data collection	5
Participants	6	(*a*) Give the eligibility criteria, and the sources and methods of selection of participants. Describe methods of follow-up<br>(*b*) For matched studies, give matching criteria and number of exposed and unexposed	5-6
Variables	7	Clearly define all outcomes, exposures, predictors, potential confounders, and effect modifiers. Give diagnostic criteria, if applicable	6-7
Data sources/ measurement	8*	For each variable of interest, give sources of data and details of methods of assessment (measurement). Describe comparability of assessment methods if there is more than one group	Table 1 Supp 3
Bias	9	Describe any efforts to address potential sources of bias	7
Study size	10	Explain how the study size was arrived at	7, fig 2
Quantitative variables	11	Explain how quantitative variables were handled in the analyses. If applicable, describe which groupings were chosen and why	Supp 3
Statistical methods	12	(*a*) Describe all statistical methods, including those used to control for confounding<br>(*b*) Describe any methods used to examine subgroups and interactions<br>(*c*) Explain how missing data were addressed<br>(*d*) If applicable, explain how loss to follow-up was addressed<br>(*e*) Describe any sensitivity analyses	7
**Results**			
Participants	13*	(a) Report numbers of individuals at each stage of study—eg numbers potentially eligible, examined for eligibility, confirmed eligible, included in the study, completing follow-up, and analysed<br>(b) Give reasons for non-participation at each stage<br>(c) Consider use of a flow diagram	7, fig 2
Descriptive data	14*	(a) Give characteristics of study participants (eg demographic, clinical, social) and information on exposures and potential confounders<br>(b) Indicate number of participants with missing data for each variable of interest<br>(c) Summarise follow-up time (eg, average and total amount)	7-8, tab 2
Outcome data	15*	Report numbers of outcome events or summary measures over time	7-9

Main results	16	(*a*) Give unadjusted estimates and, if applicable, confounder-adjusted estimates and their precision (eg, 95% confidence interval). Make clear which confounders were adjusted for and why they were included<br>(*b*) Report category boundaries when continuous variables were categorized<br>(*c*) If relevant, consider translating estimates of relative risk into absolute risk for a meaningful time period	7-9<br>tab 3<br>tab 4
Other analyses	17	Report other analyses done—eg analyses of subgroups and interactions, and sensitivity analyses	Supp 4-5
**Discussion**			
Key results	18	Summarise key results with reference to study objectives	9-10
Limitations	19	Discuss limitations of the study, taking into account sources of potential bias or imprecision. Discuss both direction and magnitude of any potential bias	9-12
Interpretation	20	Give a cautious overall interpretation of results considering objectives, limitations, multiplicity of analyses, results from similar studies, and other relevant evidence	9-12
Generalisability	21	Discuss the generalisability (external validity) of the study results	9-12
**Other information**			
Funding	22	Give the source of funding and the role of the funders for the present study and, if applicable, for the original study on which the present article is based	1, 13

*Give information separately for exposed and unexposed groups.

Note: An Explanation and Elaboration article discusses each checklist item and gives methodological background and published examples of transparent reporting. The STROBE checklist is best used in conjunction with this article (freely available on the Web sites of PLoS Medicine at http://www.plosmedicine.org/, Annals of Internal Medicine at http://www.annals.org/, and Epidemiology at http://www.epidem.com/). Information on the STROBE Initiative is available at http://www.strobe-statement.org.
